# A new application of the phase-field method for understanding the mechanisms of nuclear architecture reorganization

**DOI:** 10.1007/s00285-016-1031-3

**Published:** 2016-05-30

**Authors:** S. Seirin Lee, S. Tashiro, A. Awazu, R. Kobayashi

**Affiliations:** 1 Department of Mathematical and Life Sciences, Hiroshima University, Kagamiyama 1-3-1, Higashi-Hiroshima, 739-8530 Japan; 2 Research Institute for Radiation Biology and Medicine, Hiroshima University, Kasumi 1-2-3, Hiroshima, 734-8553 Japan; 3 Research Center for the Mathematics on Chromatin Live Dynamics, Hiroshima University, Kagamiyama 1-3-1, Higashi-Hiroshima, 739-8530 Japan; 4 Department of Mathematical and Life Sciences and Research Center for the Mathematics on Chromatin Live Dynamics, Hiroshima University, Kagamiyama 1-3-1, Higashi-Hiroshima, 739-8530 Japan

**Keywords:** Chromatin dynamics, Phase-field method, Pattern formation, 92B99

## Abstract

Specific features of nuclear architecture are important for the functional organization of the nucleus, and chromatin consists of two forms, heterochromatin and euchromatin. Conventional nuclear architecture is observed when heterochromatin is enriched at nuclear periphery, and it represents the primary structure in the majority of eukaryotic cells, including the rod cells of diurnal mammals. In contrast to this, inverted nuclear architecture is observed when the heterochromatin is distributed at the center of the nucleus, which occurs in the rod cells of nocturnal mammals. The inverted architecture found in the rod cells of the adult mouse is formed through the reorganization of conventional architecture during terminal differentiation. Although a previous experimental approach has demonstrated the relationship between these two nuclear architecture types at the molecular level, the mechanisms underlying long-range reorganization processes remain unknown. The details of nuclear structures and their spatial and temporal dynamics remain to be elucidated. Therefore, a comprehensive approach, using mathematical modeling, is required, in order to address these questions. Here, we propose a new mathematical approach to the understanding of nuclear architecture dynamics using the phase-field method. We successfully recreated the process of nuclear architecture reorganization, and showed that it is robustly induced by physical features, independent of a specific genotype. Our study demonstrates the potential of phase-field method application in the life science fields.

**Electronic supplementary material:**

The online version of this article (doi:10.1007/s00285-016-1031-3) contains supplementary material, which is available to authorized users.

## Introduction

In eukaryotes, the genome, where the genetic information is stored in the DNA molecule, shows a hierarchical structure, and this information is integrated into the chromosome of a cell nucleus. Although DNA is a long molecule that can be compacted, forming a highly condensed chromatin structure in the nucleus, the transcription of DNA represents a dynamic process. Studies showed that DNA represents a part of an ordered, folded structure in the cell nucleus, and that the formation of this structure is most likely tightly regulated. Chromatin fibers are formed when DNA molecule is wrapped around the histones, and transcriptionally inactivated and condensed DNA region is known as heterochromatin, while the more transcriptionally active and less condensed region is called euchromatin. Chromatin fibers consist of alternating euchromatin and heterochromatin structures that can interact with each other depending on the alterations in the cellular processes. In the interphase nuclei, the chromatin fibers of each chromosome are highly compartmentalized, and none of the domain structures interact, and this is called a chromosome territory (Chubb et al. 2002; Cremer and Cremer 2010).

**Fig. 1:**
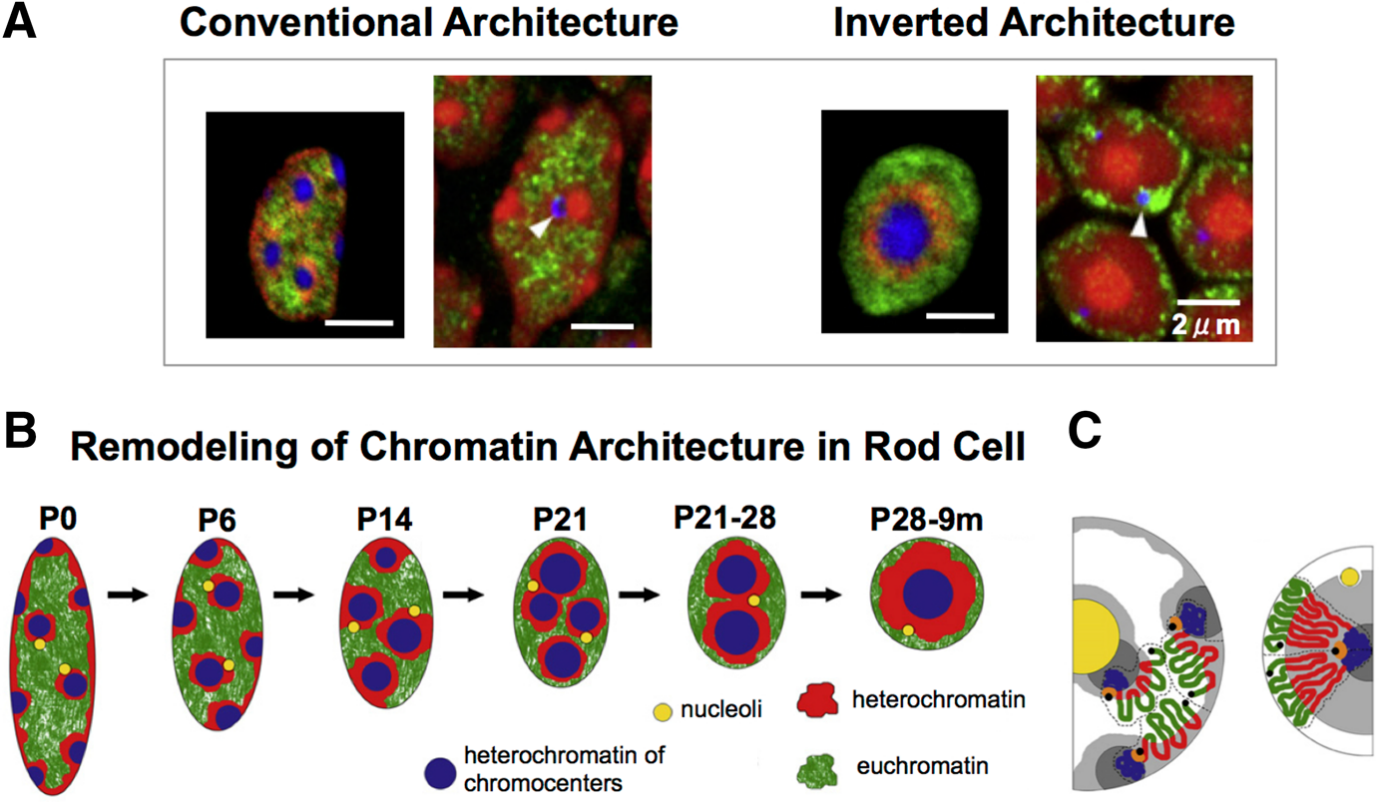
The organization of the rod cell nucleus during postnatal development (P0–P28) and in a 9-month-old mouse (9 m). **a** *Left panel* FISH, using probes for L1-rich heterochromatin (*red*), euchromatin (*green*), and chromocenters (*blue*). *Right panel* euchromatin distribution of histone H3K4me3; histone modifications (*green*), nucleoli (*blue*, *arrowheads*), and nuclear counterstaining (*red*). **b** Reorganization of the nuclear architecture. The nuclei of mouse retinas dissected at birth (P0), 6, 14, 21, and 28 days after birth (P6, P14, P21, P28, respectively), and at 9 months (9 m). When a mouse is born, the rod cell has conventional architecture. However, a few days later, the heterochromatin domains close to nuclear envelope move and fuse with neighboring heterochromatin domains, which consequently leads to the inverted architecture with a single cluster of heterochromatin domain. **c** Distribution of chromosome subregions in the nuclei with the conventional (*left*) and inverted (*right*) architecture: euchromatin (*green*, *white*), heterochromatin (*red*, *gray*), chromocenters (*blue*), nucleolus (*yellow*). The chromosomes in the nucleus with the conventional architecture relocate during the reorganization and form the inverted architecture. The diagrams in **a**–**c**) and the descriptions are adapted from Solovei et al. (2009) (colour figure online)

The positioning of heterochromatin and euchromatin is related to gene expression. In the nucleus, heterochromatin and euchromatin are spatially segregated, which contributes to the organization of nuclear function. The development of fluorescence imaging and electron microscopy revealed the spatial segregation of chromatin types into distinct subnuclear compartments (Towbin et al. 2013), and that heterochromatic clusters are not randomly distributed, but are enriched at the nuclear periphery and around the nucleoli (Gonzalez-Sandoval et al. 2013). This is called conventional architecture, and it represents a nearly universal nuclear structure, found in the majority of eukaryotic cells. In contrast to this, certain nuclei exhibit an inverted architecture, where heterochromatin is located at the center of the nucleus and euchromatin is enriched at the periphery (Fig. 1a) (Solovei et al. 2009).

Solovei et al. (2009) demonstrated that these different types of nuclear architecture are associated with different mammalian lifestyles (e.g., diurnal versus nocturnal), and are determined by the epigenetic changes. For example, the nuclear architecture of rod photoreceptor cells in the retina of diurnal mammals is typically conventional, while that in nocturnal animals is typically inverted (Solovei et al. 2013). The inverted nuclear architecture of the mouse retina rod cells is determined by the transformation of the conventional architecture, as shown in Fig. 1b. This process is accompanied by the relocation of chromosomes from their position observed in the conventional nuclear organization, like slices of pizza, and the creation of a single heterochromatin cluster (hetero-cluster) in the inverted nuclear architecture, as shown by 2D imaging (Fig. 1c). At the time of birth, the nuclei of the rod cells in mice exhibit conventional nuclear architecture. However, the distribution of chromosomes and heterochromatic clusters changes slowly, and the inverted architecture is formed during terminal differentiation. The relationships between the different types of nuclear architecture and nuclear functions are not clear. However, a previous study suggested that different types of nuclear architecture may result in the different rates of light collection efficiency, and that the inverted architecture is more suitable for this process, in comparison with the conventional nuclear architecture (Solovei et al. 2009).

Detailed analyses of this reorganization process showed that the conventional architecture is reorganized through the transformation of the nuclear shape from elliptical to circular, which is accompanied by a decrease in nuclear volume by approximately 40 % (Fig. 1b) (Solovei et al. 2009). Furthermore, structural differences between the conventional and inverted architectures can be attributed to the activity of the nuclear envelope proteins, lamin B receptor (LBR) and lamin A (Lmna), which sequentially tether peripheral heterochromatin (Solovei et al. 2013). The conventional architecture is associated with LBR or lamin A/C expression, while the expression of these molecules was not found in the cells with the inverted architecture. However, specific mechanisms mediating these events are still poorly understood, and the factors involved in the association between nuclear architecture changes and other events, such as the alterations in nuclear size or shape, are unknown. It remains unclear how the same inverted structure is consistently created in the nuclei of rod cells.

Some theoretical studies have described the spatial organization of nuclear chromatin (Finan et al. 2011; Heermann 2011; Awazu 2014; Ganai et al. 2014). The approach used in these studies is based on a model in which chromatin is represented as loops or strings, and the heterochromatin and euchromatin states are described by the differences in entropic forces or mobility at the protein structure level. These studies showed that chromatin fibers can create chromosome territories, and long-range structures composed of heterochromatin and euchromatin domains within the nucleus. However, these studies were not able to explain the specific mechanisms responsible for the formation of the conventional and inverted types of architecture. String models (Finan et al. 2011; Awazu 2014; Ganai et al. 2014) have shown that the heterochromatic domain is distributed at the nuclear periphery and not at the center of the nucleus, when no relationship between the nuclear envelope and heterochromatin is assumed, which is in contrast to the previously observed inverted architecture (Solovei et al. 2009, 2013). This indicates that the string model may not be appropriate for the determination of chromatin structure details using macroscopic models. Further macroscopic descriptions should be included in the model in order to integrate the long-range dynamics of chromatin and its molecular characteristics, and facilitate the elucidation of the key mechanisms involved in the reorganization process.

Therefore, we chose an approach that involves a higher macroscopic description by capturing chromatin as a domain, and subsequently describes the overall dynamics of chromatin based on the variations in the domain structures, since the previously described string models have shown that chromatin fibers occupy discrete territories, and that the movement of each chromatin fiber is confined within a domain. The phase-field method has been applied to a wide range of problems related to the complex dynamics of the domain interface, especially in the materials science (Provatas and Elder 2010; Takagi and Yamanaka 2012). Recently, it was used for the numerical simulation of vesicles and their bio-mechanical properties (Maitre et al. 2009; Wang and Du 2008), and was applied in cell dynamics investigations, because it simulates the complex domains of higher dimensions, such as cell shape (Akiyama et al. 2010; Shao et al. 2010). A novel method, using multi-phase-fields, applied to the studies of cell and tissue dynamics, has been proposed as well, leading to the investigations of cell division, cell adhesion, and cell sorting in higher dimensions (Nonomura 2012). Here, we chose to use the multi-phase-field method proposed by Nonomura (2012), and we developed a novel application of this method for the analysis of chromatin dynamics. Capturing chromatin within a domain can yield the information about the distribution of heterochromatin domains, as shown by the image analysis data (Solovei et al. 2009). This allowed us to focus on the elucidation of the mechanisms underlying the reorganization process in nocturnal mammals.

Two contrasting models have been used to describe chromosome territories, in which the chromatin in different chromosomes is either separated by an interchromatin compartment, or not, and in the latter model, it is able to expand into the surrounding territories (Cremer and Cremer 2010). Recently, this latter model has been supported by the findings that chromatin represents a dynamic structure, which can diffuse (Chubb et al. 2002; Gasser 2002). Chromosomes have also been found to intermingle in the interphase nuclei of human cells, and the intermingling pattern was shown to be altered depending on transcriptional activity level and chromosome condensation (Branco and Pombo 2006). Although the intermingling phase is crucial for nuclear functions, the mechanisms underlying the effects of intermingling in chromosome territories on the chromatin structures are unknown. In this study, we suggest a novel role of the intermingling phase, influencing the spatial structure of long-range chromatin distributions.

Here, we first formulate a mathematical model using the phase-field method, and show that the two types of nuclear architecture can be successfully recreated. Following this, we demonstrate how nuclear structures can be dynamically altered depending on the physical features. Afterward, we reveal that the long-range distribution of euchromatin and heterochromatin can be influenced by the size and shape of the nucleus, and finally, we discuss how the degree of intermingling between chromosome territories or between heterochromatin and euchromatin domains (He-Eu domains) plays an important role in the determination of the nuclear architecture.

**Fig. 2:**
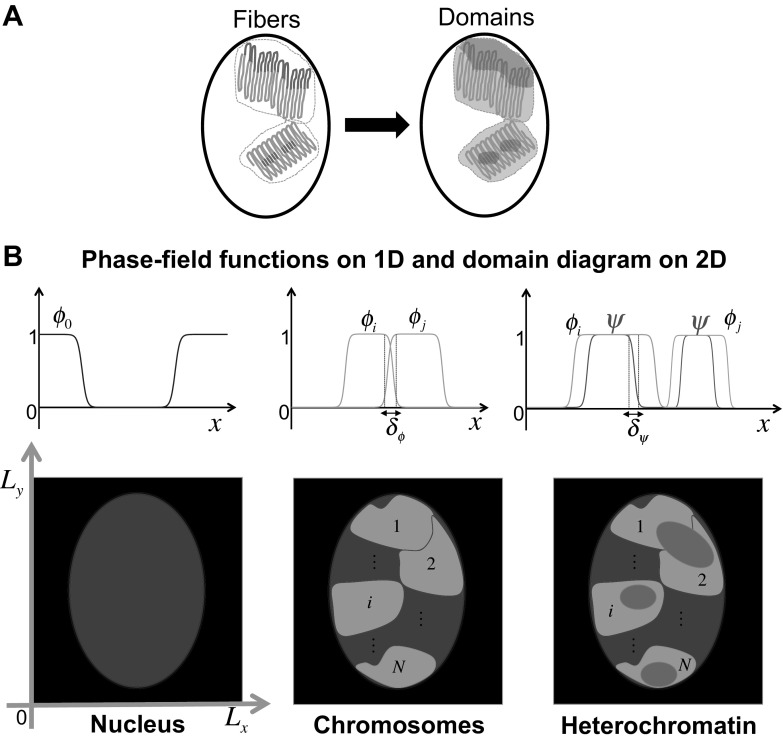
Schematic representation of the mathematical model. **a** A chromatin string is simplified so that a domain is composed of euchromatin (*green*) and heterochromatin (*red*) regions. **b** The concept underlying the phase-field method. *Blue region* represents the nuclear domain and is described by the phase-field $\phi _0$. *Green regions* are depicted by the phase-fields $\phi _1 \cdots \phi _N$, and they represent chromosome territories, while *red regions*, indicated by phase-field $\psi$, represent heterochromatin. The thickness of the intermingling region between chromosome territories is defined by $\delta _\phi$, and the thickness of the intermingling region between heterochromatin and euchromatin is defined by $\delta _\phi$ (colour figure online)

## Model formulation

Chromatin consists of alternating euchromatin and heterochromatin regions, and it is folded into chromosome territories, in which heterochromatin and euchromatin are separated. We first simplified chromatin features, using a domain composed of euchromatin and heterochromatin regions (Fig. 2a) and applied the multi-phase-field method (Nonomura 2012). We defined the three domains as follows: nucleus, chromosomes, and heterochromatin, using a continuous function called the phase-field, which describes two stable states, the present state (1) and the absent state (0) (Fig. 2b). We defined heterochromatin without distinguishing between L1-rich heterochromatin and chromocenters, in order to simplify the model further. In the definition of the three domains, the complement of the heterochromatin region in each chromosome domain represents the euchromatin domain. Therefore, an increase in the heterochromatin implies a decrease in the euchromatin in each chromosome domain. Note that we formulated a mathematical model for two dimensions of eight chromosome territories in order to compare our results directly with the results obtained previously by image analysis (Solovei et al. 2009).

### Phase-field model of nuclear architecture

We designed the model using energy functions defined by the nucleus, $\phi _0({x}, t)$, chromosome territories, $\phi _m({x}, t) ~(1\le m\le N)$, and heterochromatin, $\psi ({x}, t)$, where ${x}\in \Omega$ in ${R}^n$ and $t>0$. For the simulations, $\Omega$ is given by $L_x\times L_y$ where $L_x$ and $L_y$ are horizontal and perpendicular lengths, respectively (black square shown in Fig. 2b). *N* represents the total number of chromosomes in the nucleus and $N=8$.

First, we defined the basal free energy functions for chromosome territories and heterochromatin, according to the following equation:

$$\begin{aligned} E_0=\sum _{m=1}^{N} \int _{\Omega } \left[ \frac{\epsilon _{\phi }^2}{2}|\nabla \phi _m |^2 +g(\phi _m) \right] d{\mathbf {x}} +\int _{\Omega } \left[ \frac{\epsilon _{\psi }^2}{2}|\nabla \psi |^2 +g(\psi ) \right] d{\mathbf {x}}, \end{aligned}$$

where $\epsilon _{\phi }, \epsilon _{\psi }>0$ are gradient coefficients. Note that we defined the phase-field of the nucleus, $\phi _0$ directly, using a function satisfying $\phi _0=0$ and $\phi _0=1$ for the interior and the exterior regions of the nucleus (Fig. 2b), with a sufficiently small thickness of the interface. Next, we defined spatial range restriction for the chromatin domains, in order to avoid biologically unfeasible events. We made three assumptions based on previous observations and results:

- ($S_1$) Each chromosome is restricted in its nuclear domain (Alberts et al. 2002),
- ($S_2$) No heterochromatin region can escape from the given chromosome domain (Alberts et al. 2002),
- ($S_3$) The chromosome domains are preferentially separated from each other and constitute separate territories (Cremer and Cremer 2010).

The assumptions, $S_1$–$S_3$ are described as

$$\begin{aligned} E_1= & {} \underbrace{\beta _{0}\sum _{m=1}^{N}\int h(\phi _0)h(\phi _m)d{\mathbf {x}}}_{{S_1}} +\underbrace{\beta _{\psi }\int \left[ 1-\sum _{m=1}^{N} h(\phi _m) \right] h(\psi )d{\mathbf {x}}}_{{S_2}}\\&+\underbrace{\beta _{\phi }\sum _{m\ne n}^N \int h(\phi _n)h(\phi _m)d{\mathbf {x}}}_{{S_3}}, \end{aligned}$$

where $\beta _0$, $\beta _{\psi }$, and $\beta _{\phi }$ are positive constants and indicate the intensities of domain territories. $h(\phi _m)$ is given to $h(\phi _m)=\phi _m^3(10-15\phi _m+6 \phi _m^2)$ (See Appendix for more details about *h*). $E_0$ and $E_1$ are fundamental formulations describing nuclear and chromatin domains.

In the third step, we defined chromosome and heterochromatin volumes, so that they can reflect the changes in chromatin territories. Three assumptions were made:

- ($R_1$) The nuclear space is fully occupied by chromosomes,
- ($R_2$) The chromosome can be contracted or expanded to a given volume,
- ($R_3$) Heterochromatin is converted from/to euchromatin within each chromosome.

Hara et al. (2013) showed that the chromosome condensation is affected by the number of chromosomes per nuclear size, and the reduction of nuclear size leads to the reduction of the size of the condensed chromosome. Therefore, we made the assumption $R_1$, and that the total volume of chromosomes is related to the nuclear volume. Chromosomes are condensed before a cell division and expand after cell division. However, it is unclear how the volume of the chromosome is regulated, and we simply assumed $R_2$, based on the observations. $R_3$ is assumed based on the changes of the DNA molecule transcriptional states during the differentiation. $R_1$–$R_3$ are formulated using the equation

$$\begin{aligned} E_2= & {} \underbrace{\alpha _0\left[ \int _{\Omega } [1-h(\phi _0)]dx-\sum _{m=1}^{N} V_m(t)\right] ^2}_{R_1} +\underbrace{\alpha _V\sum _{m=1}^{N} [V_m(t)-\bar{V}_m(t)]^2}_{R_2}\\&+\underbrace{\alpha _v\sum _{m=1}^{N} [v_m(t)-\bar{v}_m(t)]^2}_{R_3} \end{aligned}$$

where $\alpha _0, \alpha _V, \alpha _v>0$ are the energy intensity constants of each volume. In the first term of $E_2$, $\int _{\Omega }[1- h(\phi _0)]d\mathbf {x}$ corresponds to the nuclear volume, because $h(\phi _0=0)=0$ and $h(\phi _0=1)=1$. $R_1$ indicates that the total volume of chromosomes has a minimal energy when its equals nuclear volume. This leads to the full occupancy of the nucleus. In the $R_2$ and $R_3$ equations, $V_m(t) (1\le m \le N)$ represents the volume of *m*-th chromosome and $v_m(t) (1\le m \le N)$ is the volume of the heterochromatin in an *m*-th chromosome at time *t*, and they are given by

$$\begin{aligned} V_m(t)=\int _{\Omega } h(\phi _m)d\mathbf {x}, \qquad v_m(t)=\int _{\Omega } h(\phi _m)h(\psi ) d\mathbf {x}. \end{aligned}$$

$\bar{V}_m(t)$ and $\bar{v}_m(t)$ are the functions of the target volumes of condensed chromosomes and heterochromatin, or those that extend to and have a minimal energy at the target volume.

In the final step, we include LBR and lamin A/C, which have a role in the interactions between heterochromatin and the nuclear envelope (Solovei et al. 2013), in our model. We expressed this through the intensity of affinity between nuclear function, $\phi _0$, and heterochromatin region function, $\psi$, as follows:

$$\begin{aligned} E_3=\gamma \int \nabla h(\phi _0) \cdot \nabla h(\psi ) d\mathbf {x}, \end{aligned}$$

where $\gamma$ is a constant that determines the intensity of the affinity. Note that $\gamma >0$ reflects the preference of heterochromatin to remain at the nuclear periphery, which is interpreted as the expression of LBR and lamin A/C in the nuclear envelope and their tethering of heterochromatin to the nuclear periphery. In contrast to this, $\gamma =0$ represents the absence of LBR and lamin A/C , which leads to the lack of heterochromatin and nuclear envelope interactions. The total energy of chromatin dynamics is given as

1 $$\begin{aligned} E=E_0+E_1+E_2+E_3. \end{aligned}$$

Afterward, we determined the functional derivatives of Eq. (1) with respect to $\phi _m~(1\le m\le N)$ and $\psi$, which drives the time for the system to evolve, satisfying

$$\begin{aligned} \frac{\partial \phi _m}{\partial t}=-\mu \frac{\delta E}{\delta \phi _m}\quad {(1\le m\le N)}, \qquad \frac{\partial \psi }{\partial t}=-\mu \frac{\delta E}{\delta \psi } \end{aligned}$$

where $\mu$ is a positive constant that represents the mobility of each phase-field. The calculations of the previous equations generated the reaction-diffusion model as follows:

2a $$\begin{aligned}&\mu ^{-1}\frac{\partial \phi _m}{\partial t}=\epsilon _\phi ^2 \nabla ^2 \phi _m+\phi _m(1-\phi _m)\left[ \phi _m-\frac{1}{2}-A_m\phi _m(1-\phi _m)\right] , \quad (1\le m\le N)\nonumber \\ \end{aligned}$$

2b $$\begin{aligned} \mu ^{-1}\frac{\partial \psi }{\partial t}=\epsilon _\psi ^2 \nabla ^2 \psi +\psi (1-\psi )\left[ \psi -\frac{1}{2}-B\psi (1-\psi )\right] , \end{aligned}$$

where $A_m$ and *B* are given as the functions of $V_m(t), \bar{V}_m(t), v_m(t), \bar{v}_m(t), h(\phi _0(\mathbf {x}, t)), h(\phi _m(\mathbf {x}, t))$ and $h(\psi (\mathbf {x}, t))$. The reaction-diffusion system (2) describes the interface of chromosome territories and heterochromatin domains that changes their dynamics, depending on $A_m$ and *B*, respectively. We non-dimensionalized the model (2) for time scale, *T*, and spatial scale, *L*, incorporating $t=T \tilde{t}$ and $\mathbf {x}=L\mathbf {\tilde{x}}$ into the model (2). The mobility constant, $\mu$, is defined by

$$\begin{aligned} \mu =T^{-1}, \end{aligned}$$

and set

$$\begin{aligned} \varepsilon ^2_{\phi }=\epsilon ^2_{\phi }L^{-2}, ~~ \varepsilon ^2_{\psi }=\epsilon ^2_{\psi }L^{-2}. \end{aligned}$$

We then obtained the following system by removing the tilde:

3a $$\begin{aligned}&\frac{\partial \phi _m}{\partial t}=\varepsilon _\phi ^2 \nabla ^2 \phi _m+\phi _m(1-\phi _m)\left[ \phi _m-\frac{1}{2}-A_m\phi _m(1-\phi _m)\right] , \quad (1\le m\le N)\nonumber \\ \end{aligned}$$

3b $$\begin{aligned} \frac{\partial \psi }{\partial t}=\varepsilon _\psi ^2 \nabla ^2 \psi +\psi (1-\psi )\left[ \psi -\frac{1}{2}-B\psi (1-\psi )\right] . \end{aligned}$$

In the model, we assumed that the size and shape of the nucleus change independently of chromatin states, and the phase-field $\phi _0$ is set as an independent function, describing the nucleus independently of the other phase-field functions.

#### Conventional architecture

The conventional architecture is the primary structure in the majority of eukaryotic cells and it is evolutionary conserved from unicellular to multicellular organisms. Because the conventional architecture is formed by the chromosome uncoiling during the initial stage immediately after cell division, we assumed that the target volumes of chromosomes constant and that the rate of heterochromatin conversion at each chromosome is not likely to change significantly. Therefore, we set $\bar{V}_m (t)=\bar{V}_m (>0$, constant) and $\bar{v}_m(t)=\bar{v}_m (>0,$ constant) in $E_2$, described the conventional architecture model by system (3), with

$$\begin{aligned} A_m= & {} 60\alpha _V(V_m -\bar{V}_m)+60\alpha _v(v_m-\bar{v}_m)h(\psi ) -60\alpha _0\left[ \int [1-h(\phi _0)]-\sum _{m=1}^N V_m \right] \\&+30\beta _0 h(\phi _0)+30\beta _\phi [\chi -h(\phi _m)] -30\beta _\psi h(\psi ),\\ B= & {} 60\alpha _v\sum _{m=1}^{N} \left[ (v_m-\bar{v}_m)h(\phi _m)\right] +30\beta _{\psi }(1-\chi )-30\gamma \nabla ^2 h(\phi _0), \end{aligned}$$

where $$\chi =\sum _{m=1}^N h(\phi _m)$$.

#### Inverted architecture

The inverted architecture is formed by the reorganization of conventional architecture during terminal differentiation, accompanied by a decrease in nuclear and chromosome volumes (Solovei et al. 2009). Therefore, the target volume of chromosome was set so that $\bar{V}_m(t)=r \bar{V}_m~(r\in (0, 1))$ in $E_2$. In contrast to this, nuclear transcriptional activity is likely to be lower after the final cell division and during terminal differentiation, and therefore, the target volume of the *m*-th heterochromatin at time *t* in the *m*-th chromosome was set as $\bar{v}_m(t)=V_m(t)\rho _m(t)$, where $\rho _{m}(t)$ represents the rate of change from euchromatin to heterochromatin at the *m*-th chromosome. We assumed that the heterochromatin conversion rate increases, which is reflected by $\rho _m(t)$, upon choosing an increasing monotone function. We used a sigmoid function described by

5 $$\begin{aligned} \rho _{m}(t)=\rho _m(0)+\frac{\bar{\rho }_{m}t}{t+\alpha _1\exp (-\alpha _2 (t-t^*))}, \end{aligned}$$

where $\rho _m(0)$ is $v_m(0)/V_m(0)$, and $\alpha _1, ~\alpha _2$ and $t^*$ are positive constants, $\bar{\rho }_m$ is the increasing rate of $v_m/V_m$, and $\rho _m(0)+\bar{\rho }_m$ attains the maximal rate of $v_m/V_m$. The model for the inverted architecture is given by system (3) with

$$\begin{aligned} A_m= & {} 60\alpha _V(V_m -r\bar{V}_m)+60\alpha _v(v_m-\rho _{m} V_m)(h(\psi )-\rho _{m})-60\alpha _0\\&\times \left[ \int [1-h(\phi _0)]-\sum _{m=1}^N V_m \right] \\&+30\beta _0 h(\phi _0)+30\beta _\phi [\chi -h(\phi _m)] -30\beta _\psi h(\psi ),\\ B= & {} 60\alpha _v\sum _{m=1}^{N} \left[ (v_m-\rho _{m} V_m)h(\phi _m)\right] +30\beta _{\psi }(1-\chi )-30\gamma \nabla ^2 h(\phi _0), \end{aligned}$$

where $$\chi =\sum _{m=1}^N h(\phi _m).$$

### Extent of the intermingling of territories

The phase-field approach assumes that the dynamics of the interface connecting the two states determines the dynamics of chromosome territories and heterochromatin. This implies that the thickness of the interface can be reinterpreted as the intermingling between chromosome territories or the intermingling between heterochromatin and euchromatin domains (He-Eu domains). We obtained the measure of intermingling directly by calculating the thickness of the interface.

A novel translation for the phase-fields $\phi _1, \ldots , \phi _m, \psi$ in Eq. (3) is defined by translating the phase-fields of chromatin as the relative DNA content (%). Therefore, $\phi _m=1$ and $\psi =1$ indicate the maximum density state of euchromatin and heterochromatin at the *m*-th chromosome, meaning, the highly condensed state. If we assume that the conditions $S_2$ and $S_3$ in $E_1$ are not too strong or too weak, we find that the phase-fields overlap in the interface region, which satisfies $0<\phi _1, \ldots , \phi _m, \psi <1$ for fixed $\beta _\psi$ and $\beta _\phi$. That is, the scale of intermingling of chromosome territories or He-Eu domains is determined by the interface thickness. Following this, the extent of intermingling of each phase-field, $\delta _\phi$ and $\delta _\psi$, can be explicitly calculated (Takagi and Yamanaka 2012), as follows:

7 $$\begin{aligned} \delta _{\phi }=4\sqrt{2}\epsilon _{\phi }\tanh ^{-1}(1-2\lambda ), \qquad \delta _\psi =4\sqrt{2}\epsilon _{\psi }\tanh ^{-1}(1-2\lambda ) \end{aligned}$$

where $\lambda$ is a constant that defines the interface region, so that $\lambda \le \phi _1, \ldots , \phi _m, \psi \le 1-\lambda$. Here, we mostly chose the value $\lambda =0.15$. The scale of $\epsilon _\phi$ and $\epsilon _\psi$ defines the scale of intermingling in our model.

### Parameters and numerical methods

The estimation of all parameters of the model from experimental data is difficult, because the reports describing chromatin dynamics are not numerous. We typically performed simulations with a non-dimensionalized system and then verified representative parameters by estimating dimensional scales through the comparisons of qualitative dynamics and previously obtained temporal data about the reorganization process (Solovei et al. 2009).

In system (3), we defined the mobility of phase-fields as $\mu =T^{-1}$. That is, we can determine $\mu$ by estimating the time scale, *T*. For this, we directly compared qualitative dynamics of a non-dimensionalized system (3) with the previously reported experimental data (Solovei et al. 2009) (Fig.  1). We were then able to estimate *T* from $t=T \tilde{t}$, which consequently allowed the determination of $\mu$. With representative parameters set, *T* was estimated to be 5 h, and we obtained $\mu =1/5~(h^{-1})$.

Additionally, we were able to estimate the spatial scale directly from the experimental images obtained previously by Solovei et al. (2009), in which the size of the nucleus of the rod cell in two dimensions was determined to be approximately 4–5 $\upmu$m (*x*-axis) and 6–8 $\upmu$m (*y*-axis) in P0 cells. We chose 5 $\upmu$m as the *x*-axis diameter and and 8 $\upmu$m as the *y*-axis diameter of the elliptic nucleus, and used $L_x=6 ~\upmu$ m for the *x*-axis and $L_y=9~\upmu$m for the *y*-axis in space for numerical simulations. In the non-dimensional system, we used $1.2 \times 1.8$ square, so that $L= 5~\upmu$m allows $L_x=6~\upmu$m for the *x*-axis and $L_y=9~\upmu$m in the dimensional system.

With *T*, $\mu$ and *L* known, we are able to confirm whether $\varepsilon _\phi ^2=\epsilon _\phi ^2 L^{-2}$ and $\varepsilon _\psi ^2=\epsilon _\psi ^2 L^{-2}$ fall within a reasonable range of parameters in the dimensional system (3). First, we scaled the dimensionless parameters that we used for simulations with *T*, $\mu$ and *L*, and obtained the dimensional values shown in Table 1. $\mu \epsilon _\phi ^2$ and $\mu \epsilon _\psi ^2$ from Eq. (3) are considered the diffusion coefficients of euchromatin and heterochromatin. Chromatin mobility in mammalian nuclei is reported not to exceed 0.4 $\upmu$m for time periods of over 1 h (Abney et al. 1997), and therefore, the diffusion coefficients of chromatins can be estimated on a scale of less than $0.16~\upmu \mathrm{{m}}^2/\mathrm{{h}}$. The parameter values in Table 1 show that the diffusion rates of chromatin we used are $\mu \epsilon _\phi ^2 \in [0.9 \times 10^{-3}, ~3.7\times 10^{-3}~\upmu \mathrm{{m}}^2/\mathrm{{h}}]$ for euchromatin and $\mu \epsilon _\psi ^2 \in [0.98\times 10^{-3}, ~3.92\times 10^{-3}~\upmu \mathrm{{m}}^2/\mathrm{{h}}]$ for heterochromatin, since both of these are sufficiently smaller than $0.16 ~\upmu \mathrm{{m}}^2/\mathrm{{h}}$.

We also estimated the extent of intermingling with a similar approach and obtained $\delta _\psi$ $[0.343,~0.687~\upmu \mathrm{{m}}]$ = $[343,~687 ~\mathrm{{nm}}]$ and $\delta _\phi [0.329, ~0.667~\upmu \mathrm{{m}}]=[329, ~667~\mathrm{{nm}}]$ from Eq. (7). This scale is also considered to be in a feasible range, because the length of chromatin fibers is thought to be approximately 300 nm in eukaryotic genomes (Rosa and Everaers 2008).

The representative dimensional/nondimensional parameters are presented in Table 1, except for $\alpha _0, ~\alpha _V, ~\alpha _v,$ $~\beta _0, ~\beta _\phi , ~\beta _\psi$, which are the same in both non-dimensional and dimensional systems, so that $\alpha _0=25/6, ~\alpha _V=10/6, ~ \alpha _v=20/3, ~\beta _0=5/3,~\beta _\phi =1$, and $\beta _\psi =2/3$.

**Table 1:** Parameters details

Dimensionless		Dimensional	
Symbol	Value	Symbol	Value
$*$	$*$	*L*	5 $\upmu$m
$*$	$*$	*T*	5 h
$*$	$*$	$\mu$	0.2 $h^{-1}$
$L_x$	1.2	$L_x$	6 $\upmu$m
$L_y$	1.8	$L_y$	9 $\upmu$m
$\bar{t}$	1.0	*t*	5*h*
$\varepsilon _\phi ^2$	$[1.8 \times 10^{-4}, ~ 7.4\times 10^{-4}]$	$\epsilon _\phi ^2$	$[4.5 \times 10^{-3}, ~ 18.5\times 10^{-3}](\upmu \mathrm{{m}}^2)$
$\varepsilon _\psi ^2$	$[1.96\times 10^{-4}, ~ 7.84\times 10^{-4}]$	$\epsilon _\psi ^2$	$[4.9 \times 10^{-3}, ~ 19.6\times 10^{-3}](\mu \mathrm{{m}}^2)$
$\gamma$	0.0022/3	$\gamma$	0.55 $\mu \mathrm{{m}}^2$

We solved the Eq. (3) using an explicit numerical algorithm written in C programing language. In order to solve the Laplacian terms in the model, we used the standard method by applying twice the central difference operator (Morton and Mayers 1994). Therefore, the numerical simulation does not blow up if (diffusion constant)$\times$[(timestep)/(gridsize of *x*-axis)$^2$+ (timestep)/(gridsize of *y*-axis)$^2$]$\le 1/2$, with sufficiently small time steps. We took a time step of $6\times 10^{-4}$ for the grid size $6\times 10^{-3}$ of *x*- and *y*-axis. The ratio of minimal interface width to the chosen grid size was approximately 26.33, and the grid size was sufficiently small not to influence the movement of interfaces.

## Simulation results

When presenting the simulation results, nucleus was depicted in blue, chromosome territories in green, and heterochromatin in red (Fig. 2b). The complementary region of heterochromatin in each green domain is implicitly euchromatin. We first generated the conventional architecture and then show the inverted architecture through the regeneration of the reorganization process. Following this, we explored the mechanisms underlying the reorganization process and the pattern formation of chromatin.

### Conventional architecture

**Fig. 3:**
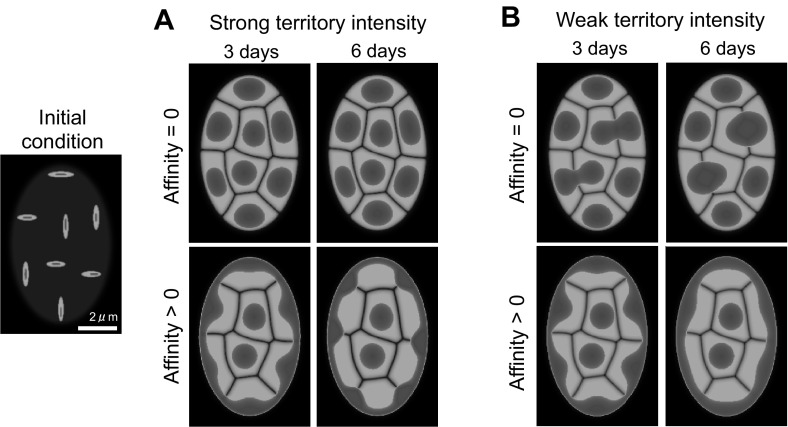
Conventional architecture following the cell division. The parameters for domain separation are chosen differently in **a** and **b**. **a** Shows stronger territory intensities compared with those used in **b**. Affinity $=0$ indicates the condition in which heterochromatin is completely independent of the nuclear envelope, and affinity $>0$ indicates that heterochromatin is tethered either to LBR or lamin A/C in the nuclear envelope. $\alpha _V=\alpha _v=2, ~\beta _0=5/3, ~\gamma =0$ or $0.0022/3, ~\varepsilon _{\phi }^2=0.0002, ~\varepsilon _{\psi }^2=0.0004,$ and $\bar{\mu }_0=\bar{\mu }_\phi =\bar{\mu }_\psi =1$. $(\beta _\phi , ~\beta _\psi )=(8/3, 8/3)$ in A and (2, 2/3) in B. $\bar{V}_m=$Nuclear volume/*m* and $\bar{v}_m=V_m\times [0.23,~0.28]$, where $m=8$

The highly condensed chromosome in the early stage of mitosis or meiosis becomes uncoiled after cell division, and the chromatin in the mouse rod cells has the conventional architecture at the time of birth. In order to regenerate the conventional architecture, we initiated simulations under the initial condition, as shown in Fig. 3, and compared the results obtained by changing the parameters that control the intensity of domain territories and the affinity between heterochromatin and the nuclear envelope. A high intensity of domain territory indicates that chromosome territories are strongly confined. Zero affinity indicates that there are no interactions between heterochromatin and the nuclear envelope, but a positive affinity value represents a tethering of heterochromatin to LBR or lamin A/C on the nuclear envelope.

The results are shown in Fig. 3a, b. We first demonstrate that the conventional architecture is obtained with the positive affinity. In contrast to this, when there is no affinity, heterochromatin domains are confined completely to the center of each chromosome if the domain separation is strong, or they are fused with adjacent heterochromatin if the domain separation is weak. The obtained results indicate that positive affinity is a required condition for the localization of heterochromatin at the nuclear envelope. In particular, 6 days after the cell division, the difference in territory intensities results in slightly different types of conventional architecture. In Fig. 3a, it is shown that heterochromatin accumulates at the territories between chromosomes instead of in the region of the nuclear envelope. Therefore, small domes are generated between chromatin territories. In Fig. 3b, however, heterochromatin is shown to be distributed almost homogeneously along the nuclear envelope.

The division of rod cells in the mouse ceases 5 days after birth (Solovei et al. 2009), and, the conventional architecture of P0 in Fig. 1 is likely last less than 5 days. We chose to use data from day 3 as the initial condition for inverted architecture simulations, since it is not sensitive to the parameter of territory intensity. Our simulation results indicate that the affinity between heterochromatin and the nuclear envelope is crucial for the formation of the conventional architecture, which confirms that the expression of LBR and lamin A/C is indispensable for the generation of conventional nuclear organization, as previously shown (Solovei et al. 2013).

**Fig. 4:**
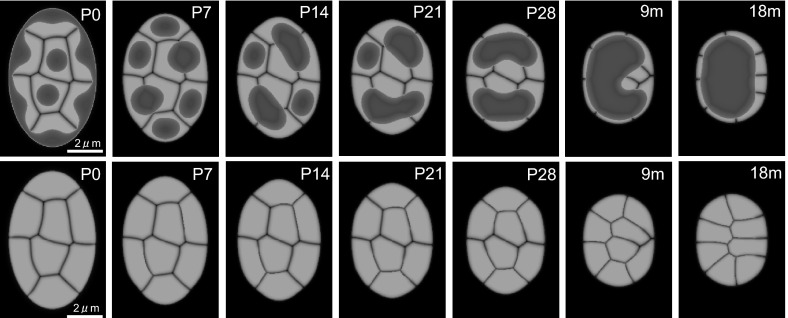
Reorganization of the nuclear architecture. The figures in the *second row* are displayed without the heterochromatin shown in the figures displayed in the first row, in order to show the details of chromosomal rearrangements. The nucleus decreases to 60 % of its original size and acquires a circular shape. $\alpha _0=25/6, ~\alpha _V=10/6, ~ \alpha _v=20/3, ~\beta _0=5/3,~\beta _\phi =1, ~\beta _\psi =2/3, ~\gamma =0.0, ~\varepsilon _\phi ^2=0.0002,$ and $\varepsilon _\psi ^2=0.0006$. For $\rho _m(t)$, $\alpha _1=120, ~\alpha _2=0.03, ~T=120$ for $\phi _1\cdots \phi _6$ and $\alpha _1=150, ~\alpha _2=0.03, ~T=150$ for $\phi _7,~\phi _8$. $(\bar{\rho }_1, ~\bar{\rho }_2, ~\bar{\rho }_3, ~\bar{\rho }_4, ~\bar{\rho }_5, ~\bar{\rho }_6, ~\bar{\rho }_7, ~\bar{\rho }_8)=(0.35, 0.4, 0.4, 0.35, 0.15, 0.15, 0.35, 0.35)$

**Fig. 5:**
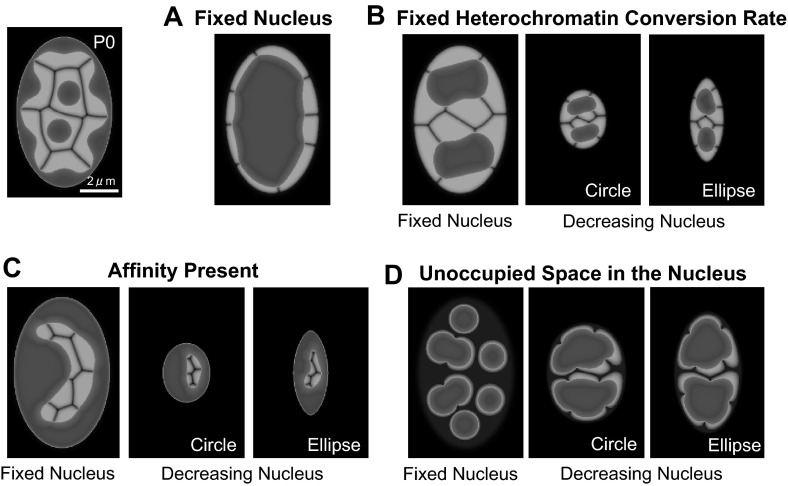
Mechanism leading to the generation of a single hetero-cluster inverted architecture. The patterns were generated from P0. **a** Nuclear size and shape are fixed under conditions (3)–(5), defined in the text. The parameters used are the same as in Fig. 4, except for $\rho _m(t)=0$. **b** The heterochromatin conversion rate is fixed for a fixed-sized nucleus, and circular and elliptical nuclei that decrease in size (80 % cut-off) under conditions (4)–(5), defined in the text. The parameters used are the same as in Fig. 4, except for $(\bar{\rho }_1, ~\bar{\rho }_2, ~\bar{\rho }_3, ~\bar{\rho }_4, ~\bar{\rho }_5, ~\bar{\rho }_6, ~\bar{\rho }_7, ~\bar{\rho }_8)=(0.0, 0.0, 0.0, 0.0, 0.0, 0.0, 0.0, 0.0)$. **c** The affinity between the nuclear envelope and heterochromatin is present for a fixed-sized nucleus and circular and elliptical nuclei that decrease in size (80 % cut-off) under conditions (3) and (5), defined in the text. The parameters used are the same as in Fig. 4, except for $\gamma =0.022$. **(d)** The unoccupied space in the nucleus is considered a fixed nucleus and circular and elliptical nuclei that decrease in size (40 % cut-off) under conditions (3) and (4), defined in the text. The final volumes of euchromatin and heterochromatin decrease to approximately 49 % compared with P0 volumes, in each panel. The parameters used are the same as in Fig. 4

### Inverted architecture and reorganization process

We first demonstrate the successful regeneration of the reorganization process (Fig. 4; Supplementary Movie S1), as proposed in Fig. 1b. Heterochromatin dynamics and the total dynamics of chromosome rearrangement are precisely regenerated so that randomly distributed chromosomes in the conventional architecture are rearranged as suggested in Fig. 1c. The chromosome positions before 28 days (P28) remain virtually unchanged, although heterochromatin clusters fuse and form two separate clusters. However, once heterochromatin forms a single cluster, the rearrangement of chromosome territory and the inversion of the nuclear architecture are achieved. Note that the temporal scale of qualitative dynamics in our simulation is very similar to that obtained by the experimental observations. The terminal fusion (P28–9 m) was shown to last longer than the fusion stage from P0 to two clusters (P0–P28). A comparison of Figs. 1b and 4 shows how time and spatial dynamics in our simulation coincide with the previous experimental observations (Solovei et al. 2009).

#### Mechanism of single hetero-cluster inverted architecture generation

The successful regeneration of the reorganization is based on the following five assumptions:

1. The size of the nucleus decreases.
2. The shape of the nucleus changes from elliptical to circular.
3. The rate of conversion of heterochromatin to euchromatin increases.
4. There is a lack of affinity between the nuclear envelope and heterochromatin.
5. The nucleus is fully occupied by chromosomes.

We evaluated these five conditions sequentially, in order to define which conditions are indispensable for the induction of the single hetero-cluster inverted architecture generation. First, we fixed the size of the nucleus, in order to determine whether a decreased nuclear size and circular shape are indispensable. Surprisingly, the obtained data, presented in Fig. 5a indicated that the increase in the heterochromatin conversion rate is sufficient for the formation of the inverted architecture. This demonstrated that the change to a circular nuclear shape is not crucial for this process. However, when the increase in the heterochromatin conversion rate is insufficient, the inverted architecture formation, with a single heterochromatin cluster, cannot be achieved, even when the decrease in the size of the nucleus is sufficiently small, regardless of its shape (Fig. 5b). When the heterochromatin conversion rate is constant, it is possible for the heterochromatin domain to fuse, so that the number of clusters decreases. However, a single cluster of heterochromatin cannot form without a sufficient increase in heterochromatin conversion rate.

Next, we assessed the effect of the affinity between the nuclear envelope and heterochromatin. The data presented in Fig. 5b confirm that the absence of affinity is indispensable for the formation of inverted architecture, regardless of nuclear size and shape. When the affinity between the nuclear envelope and heterochromatin exists, it remains at the nuclear periphery and the inverted architecture is never formed.

Figure 5d shows that the inter-chromatin compartment and the space between the chromosome and the nuclear envelope must be completely occupied, in order to induce the formation of a single heterochromatin cluster. When the total volume occupied by chromosomes is insufficient to fill the nucleus, chromosome territories are separated more strictly and heterochromatin is unable to fuse, regardless of the nuclear shape. Therefore, chromosome volumes must decrease to a size no smaller than that of the nuclear volume, in order for the single hetero-cluster inverted architecture to form.

A decrease in nuclear size and nuclear deformation are not indispensable for the reorganization process, but the increase in the rate of conversion of heterochromatin to euchromatin, the absence of affinity, and the absence of unoccupied space in the nucleus are crucial for the induction of the transformation from the conventional to the inverted architecture.

**Fig. 6:**
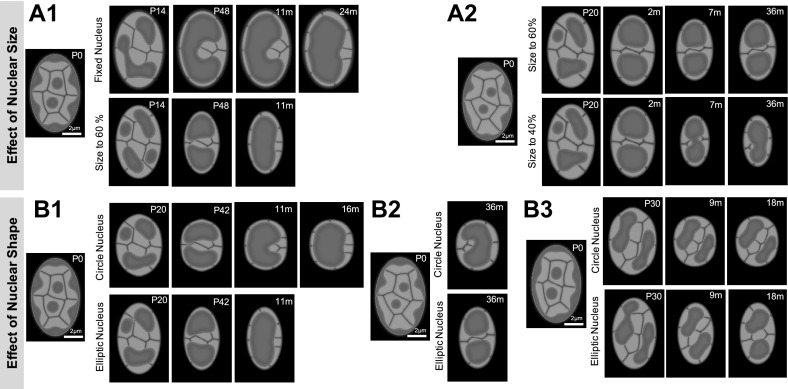
Influence of nuclear size and shape on nuclear pattern. The *upper panels* show the effect of nuclear size on the reorganization time scale (**A1**) and the final pattern (**A2**). The lower panels show the effect of nuclear shape on reorganization time scale (**B1**), final pattern (**B2**), and the rearrangement of chromosome territories (**B3**). The detailed parameters are as follows: *A1* The same as in Fig. 4, except $\rho _m(t)=0$ in the first row of figures. **A2** $\alpha _0=25/6, ~\alpha _V=5/3, ~ \alpha _v=20/3, ~\beta _0=5/3,~\beta _\phi =1, ~\beta _\psi =2/3, ~\gamma =0.0, \varepsilon _\phi ^2=0.0003,$ and $\varepsilon _\psi ^2=0.0006$. For $\rho _m(t)$, $\alpha _1=120, ~\alpha _2=0.03, ~T=120$ for $\phi _1\cdots \phi _6$ and $\alpha _1=80, ~\alpha _2=0.03, ~T=80$ for $\phi _7,\phi _8$. $(\bar{\rho }_1, ~\bar{\rho }_2, ~\bar{\rho }_3, ~\bar{\rho }_4, ~\bar{\rho }_5, ~\bar{\rho }_6, ~\bar{\rho }_7, ~\bar{\rho }_8)=(0.35, 0.15, 0.2, 0.35, 0.15, 0.15, 0.6, 0.6)$. (B1) The same as in Fig. 4. (B2) The same as in (A2). (B3) $\alpha _0=25/6, ~\alpha _V=5/3, ~ \alpha _v=20/3, ~\beta _0=5/3,~\beta _\phi =1, ~\beta _\psi =2/3, ~\gamma =0.0, ~ \varepsilon _\phi ^2=0.0002,$ and $\varepsilon _\psi ^2=0.0004$. For $\rho _m(t)$, $\alpha _1=150, ~\alpha _2=0.03, ~T=150$ for $\phi _1\cdots \phi _6$ and $\alpha _1=250, ~\alpha _2=0.03, ~T=250$ for $\phi _7,\phi _8$. $(\bar{\rho }_1, ~\bar{\rho }_2, ~\bar{\rho }_3, ~\bar{\rho }_4, ~\bar{\rho }_5, ~\bar{\rho }_6, ~\bar{\rho }_7, ~\bar{\rho }_8)=(0.1, 0.1, 0.0, 0.0, 0.2, 0.1, 0.2, 0.2)$

**Fig. 7:**
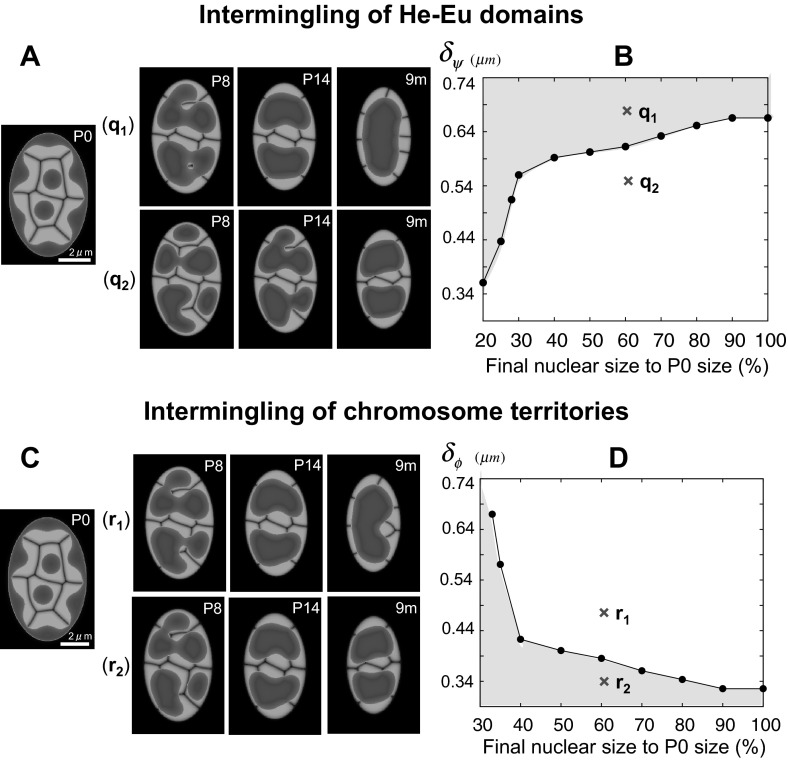
The influence of intermingling thickness of He-Eu domains ($\delta _\psi$) and chromosome territories ($\delta _\phi$) on The reorganization of chromatin architecture. **a**–**b** $\delta _\psi$ varies for fixed $\delta _\phi =0.345~\upmu$m. q$_1=0.664~\upmu$m and q$_2=0.537~\upmu$m. **c**–**d** $\delta _\phi$ varies for fixed $\delta _\psi =0.646~\upmu$m. r$_1=0.489~\upmu$ m and r$_2=0.342~\upmu$m. The nuclear size decreases to 60 % in **a** and **c**. The points in **b** and **d** represent the minimal and maximum values, $\delta _\psi$ and $\delta _\phi$, respectively, for the formation of the single hetero-cluster inverted pattern. The single hetero-cluster inverted architecture is formed in the gray region. More specifically, the parameters are $\alpha _0=25/6, ~\alpha _V=5/3, ~ \alpha _v=20/3, ~\beta _0=5/3,~\beta _\phi =1, ~\beta _\psi =2/3, ~\gamma =0.0,$ and $\varepsilon _0=0.00002$. For the heterochromatin function, $\rho _m(t)=\rho _m(0)+\bar{\rho }_m t/(10+t)$ was used with $(\bar{\rho }_1, ~\bar{\rho }_2, ~\bar{\rho }_3, ~\bar{\rho }_4, ~\bar{\rho }_5,$ $~\bar{\rho }_6, ~\bar{\rho }_7, ~\bar{\rho }_8)=(0.05, 0.3, 0.3, 0.05, 0.15, 0.15, 0.3, 0.3)$. In **a** and **b**, $\varepsilon _\phi ^2=0.0002$ is fixed and $\varepsilon _\psi ^2$ varies from 0.000196 to 0.000784. $\varepsilon _\psi ^2=0.000784, 0.000484$ are chosen in $q_1$ and $q_2$, respectively. In **c** and **d**, $\varepsilon _\psi ^2=0.0007$ is fixed and $\varepsilon _\phi ^2$ varies from 0.00018 to 0.00074. $\varepsilon _\psi ^2=0.0004, 0.000196$ are chosen in $r_1$ and $r_2$, respectively

#### Nuclear size and deformation effects on nuclear patterns

In the previous section, we have found that the deformation of nuclear size and shape were not indispensable for the formation of the single hetero-cluster inverted architecture. However, it was reported that the size of the rod cell nuclei differs by approximately 40 % between P0 and 9 m and the nuclear shape changes from an elliptical to a circular (Solovei et al. 2009). Therefore, we investigated how these two features influence chromatin dynamics during the reorganization.

First, we found that the nuclear size and shape influence the time scale of reorganization (Fig.  6A1 and B1). The formation of the same type of inverted patterns in the fixed-domain case required almost twice as long to complete, compared with the formation in a 40 % smaller nucleus. Similarly, the formation in a circular nucleus lasts longer than in an elliptical nucleus, because the distance that two central chromosomes need to cover in order to reach the nuclear periphery is longer in larger and circular nuclei compared with that in the smaller and elliptical nuclei. The speed of the movement of the interface between chromatin territories is similar.

Nuclear size and shape influence the final pattern of the inverted architecture as well (Fig. 6A2 and B2). The decrease in the nuclear size leads to a decrease in the absolute distance between heterochromatin clusters, which influences the size of intermingling of chromosome territories or He-Eu domains. For example, a circular nucleus likely facilitates the formation of a single heterochromatin cluster, in contrast to an elliptical nucleus. At equal volumes, the diameter of a circle must be shorter than the long diameter of an ellipse, which increases the distance between heterochromatin clusters in an elliptical nucleus.

The nuclear shape significantly influences chromosomal rearrangement (Fig. 6B3). Although the two geometries minimally alter the difference between heterochromatin patterns, the position of chromosomes in an elliptical nucleus changes more dynamically compared with that in a circular nucleus.

#### Intermingling of chromosome and He-Eu domains during the formation of a single hetero-cluster nuclear architecture

In order to identify the role of intermingling on the reorganization of nuclear architecture, we focused on the extent of the intermingling between chromosome territories ($\delta _\phi$) and the extent of intermingling between a euchromatin and heterochromatin domains ($\delta _\psi$), which were directly calculated in our model.

We investigated how the inverted architecture is changed by the extent of intermingling (Fig. 7). First, the extent of intermingling exerts a significant effect on the terminal pattern of inverted architecture, and the two regions of intermingling, $\delta _\phi$ and $\delta _\psi$, play different roles. In the case of He-Eu domains (Fig. 7a), the larger intermingling region is observed prior to the generation of a single heterochromatin cluster. In contrast to this, the intermingling between chromosome territories shows the opposite effects (Fig. 7c), i.e., the smaller intermingling region is observed prior to generation of a single heterochromatin cluster. These observations indicate that there is a minimum value for $\delta _\phi$ and a maximum value for $\delta _\phi$ required for the formation of the single hetero-cluster inverted architecture.

Next, we focused on the relationship between the extent of intermingling and the nuclear size required for the formation of the inverted architecture (Fig. 7b, d). We identified a minimum thickness of He-Eu domains necessary for the formation of the single hetero-cluster inverted architecture, which is presented in Fig. 7b and a maximum thickness of chromosome territories necessary for the formation of the single hetero-cluster inverted architecture, presented in Fig. 7d. The obtained data suggest that the extent of intermingling of He-Eu domains needs to be expanded for the formation of the single hetero-cluster inverted architecture, when the nucleus is large. In contrast to this, the extent of intermingling of chromosome territories needs to be reduced when nucleus is large, and the single hetero-cluster inverted architecture is more likely to form when the chromosomes occupy strictly defined territories.

## Discussion

We studied two types of nuclear architecture and the process of reorganization of the photoreceptor rod cell nucleus from the conventional to the formation of inverted architecture, using mathematical modeling, with the phase-field method. Our analyses demonstrate that an increase in the rate of conversion of heterochromatin to euchromatin and the loss of affinity of the nuclear envelope for heterochromatin in the absence of both LBR and lamin A/C expression are indispensable for this reorganization. Furthermore, the extent of intermingling between chromosome territories strongly influences the formation of the single hetero-cluster inverted architecture, related to a specific nuclear size. These findings suggest that the transformation of the chromatin from euchromatin to heterochromatin state induces initially the long-range movement of the chromosome territories. Additionally, the physical features of chromosome territories or nucleus play a crucial role in the determination of chromatin dynamics.

It was shown previously that nuclear size and shape are associated with mammalian lifestyle (Solovei et al. 2009). In nocturnal mammals, a smaller and circular nucleus with a single hetero-cluster inverted architecture reduces light scattering. Here, we analyzed the direct influence of nuclear size and shape on nuclear architecture. Our results demonstrate that the size and shape of the nucleus are crucial for the determination of inverted architecture pattern, and the temporal scale of the reorganization process. This is the first theoretical study to consider the effect of nuclear deformation on chromatin reorganization.

We explored the effects of chromosome intermingling on nuclear architecture and showed that the intermingling of chromosome territories and He-Eu domains can play opposite roles in the creation of the single hetero-cluster inverted architecture, based on the nuclear size. This suggests that nuclear size can play an important role in chromatin distribution, independently of, or together with, the intermingling dynamics.

Previous studies (Awazu 2014; Finan et al. 2011; Ganai et al. 2014; Heermann 2011), employing the microscopic modeling approach to chromatin dynamics, have shown that a phase separation between heterochromatin and euchromatin exist, which leads to the formation of long-range clusters in heterochromatin. Therefore, the conversion of euchromatin to heterochromatin, and heterochromatin mobility can be investigated by analyzing heterochromatin domain dynamics. Since our model is based on a macroscopic approach, we were able to capture the overall chromatin dynamics in the nucleus, even when the detailed features of chromatin at the fiber level were not known. Many previous studies have successfully applied the macroscopic approach to gain understanding of micro-scale problems. For example, the phase separation problems in polymer solution systems, such as block copolymer dynamics, have been solved by the macroscopic approach, which does not depend on the microscopic details of polymers. This confirms that the macroscopic approach may represent a very useful tool for the facilitation of the understanding of soft matter systems (Fredrickson et al. 2002; Pinna and Zvelindovsky 2012; Yamada et al. 2004).

The details of chromatin structures and how chromatin dynamics is regulated spatially and temporally has not been completely elucidated, and our mathematical model, using a top–down approach, may help understand these structures and processes. The findings presented here suggest a potential mechanism underlying the reorganization of nuclear architecture. Although the properties of chromatin are simplified in our model, and we have made assumptions about several features of the reorganization process, the obtained results suggest that understanding the primary physical features of the nucleus may be sufficient to allow the understanding of the core mechanism of nuclear reorganization. Our study represents the first step toward the understanding of chromatin dynamics by incorporating the information about the molecular properties of chromatin with the macro level chromatin dynamics data. Furthermore, our model demonstrates the applicability of phase-field method in life science investigations.

### Electronic supplementary material

Below is the link to the electronic supplementary material.

Supplementary material 1 (mpg 1054 KB)

## Figures and Tables

**Fig. 8:**
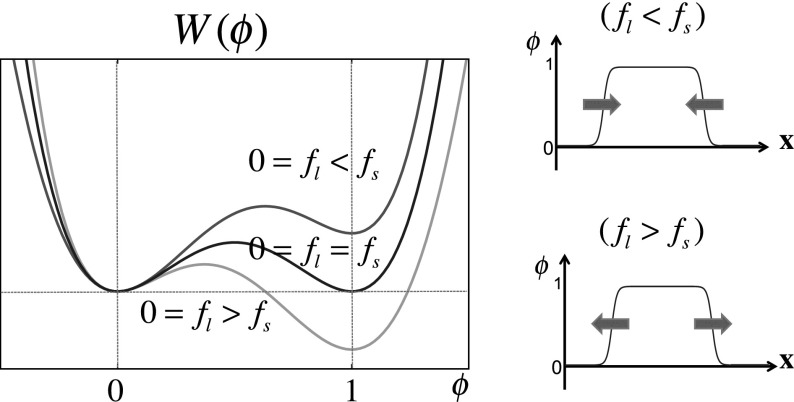
$W(\phi )$ example. The *red*, *blue*, and *green* lines in the *right panel* correspond to $f_l<f_s$, $f_l=f_s$, and $f_l>f_s$, respectively. In the panels on the *right*, the *arrows* indicate the direction in which the interfaces move when $f_l<f_s$ and $f_l>f_s$ (colour figure online)

## Appendix

The basal phase-field model describing chromatin territories is based on the following Ginzburg–Landau free energy equation (Nonomura 2012; Provatas and Elder 2010):

$$\begin{aligned} E_{basal}= \int _{\Omega } \left[ \frac{\epsilon _{\phi }^2}{2}|\nabla \phi |^2 +W(\phi ) \right] d\mathbf {x}, \end{aligned}$$

where $\epsilon _{\phi }$ is a gradient coefficient. $W(\phi )$ is given as $W(\phi )=g(\phi )+f_s h(\phi )+f_l (1-h(\phi ))$, where $g(\phi )=\frac{1}{4}\phi ^2(1-\phi )^2$, $h(\phi )=\phi ^3(10-15\phi +6 \phi ^2)$, and $f_s$ and $f_l$ are constants. The form of $h(\phi )$ is mathematically justified. The symmetric potential $g(\phi )$ is used for setting the local minima at $\phi = 0$ and $\phi = 1$ (therefore, no driving force of interface). $h(\phi )$ is used for the induction of an energetic asymmetry between $\phi = 0$ and $\phi = 1$, driving the interface while keeping $\phi = 0$ and $\phi = 1$ local minima of the energy function $W(\phi )$. Therefore, the required conditions for $h(\phi )$ are

$$\begin{aligned} h(0) = 0, h(1) = 1, h'(0) = h'(1) = 0, h''(0) = h''(1) = 0. \end{aligned}$$

$h(\phi ) = \phi ^{3} (10 - 15\phi + 6 \phi ^{2})$ is the lowest order polynomial which satisfies six previously described conditions, which is a standard choice in the phase field method.

Therefore, $W(\phi )$ describes a double-well potential which has local minimums at $\phi =0$ and 1 if $|f_s-f_l|<1/12$. The constants $f_s$ and $f_l$ correspond the free energy densities for the phase described by $\phi =1$ and $\phi =0$ By taking the functional derivative of $E_{basal}$ with respect to $\phi$, we can induce a time evolution model of $\phi$ driven by $\partial \phi /\partial t = -\mu \delta E_{basal}/\delta \phi$, as follows:

8 $$\begin{aligned} \mu ^{-1}\frac{\partial \phi }{\partial t}=\epsilon _\phi ^2 \nabla ^2 \phi +\phi (1-\phi )\left[ \psi -\frac{1}{2}-60(f_s-f_l)\phi (1-\phi )\right] , \end{aligned}$$

where $\mu$ is a positive constant. Eq. (8) provides a smooth front solution connecting the region $\phi =1$ and $\phi =0$ so that the direction of the front movement depends on *sgn*($f_s-f_l$). Formulating the terms of $f_s$ and $f_l$ using the function $h(\phi )$ is the core of the phase-field modeling.

This model gives a traveling wave solution connecting $\phi =0$ and $\phi =1$. In fact, the depth of the well of the function $W(\phi )$ is controlled by the constants, $f_s$ and $f_l$, as shown in Fig. 8. If $f_l<f_s$ ($f_l>f_s$), $\phi =0$ ($\phi =1$) region expands. In the case of $f_s=f_l$, this implies that the phase interface is stopped.
